# Editorial: Recent Advances in Basic and Translational Osteoimmunology

**DOI:** 10.3389/fimmu.2021.800508

**Published:** 2021-11-18

**Authors:** Rupesh K. Srivastava, Katharina Schmidt-Bleek, Naibedya Chattopadhyay, Massimo De Martinis, Pradyumna Kumar Mishra

**Affiliations:** ^1^ Department of Biotechnology, All India Institute of Medical Sciences (AIIMS), New Delhi, India; ^2^ Julius Wolff Institut, Berlin Institute of Health at Charité – Universitätsmedizin Berlin, Charitéplatz 1, Berlin, Germany; ^3^ Division of Endocrinology and Centre for Research in Anabolic Skeletal Targets in Health and Illness (ASTHI), CSIR-Central Drug Research Institute, Lucknow, India; ^4^ Department of Life, Health and Environmental Sciences, University of L’Aquila, L’Aquila, Italy; ^5^ Department of Molecular Biology, ICMR-National Institute for Research in Environmental Health (NIREH), Bhopal, India

**Keywords:** osteoimmunology, immune cells, bone, immunoporosis, cytokines, inflammation

The term “Osteoimmunology” was coined to describe the correlation between bone and immune cells (1). Recent findings have highlighted the link between both the bone and immune systems (2), an interrelation further supported by the common developmental niche, Bone Marrow (BM). “Immunoporosis” as an independent field under the umbrella of osteoimmunology was recently proposed in 2018 to highlight the growing importance of the immune system in Osteoporosis (3, 4). The present Research Topic on ‘Recent advances in Basic and Translational Osteoimmunology’ comprises research in the emerging field of Osteoimmunology. This Research Topic brings together 17 contributions by 116 authors from across the globe, including the U.S.A. (6), Europe (72), and Asia (38). The compilation of these recent reviews and research articles deepens understanding of the role of the immune system in various skeletal system associated pathologies.

## Role of the Adaptive Immune System in Skeletal Homeostasis

Primarily, osteoimmunology emphasizes the two-way communication between the bone and immune cells. The interrelation between the bone and immune system plays an indispensable role in the maintenance of skeletal homeostasis. The first question that comes to mind is how the immune system can contribute to the differentiation and functional activity of bone cells. Furthermore, Th17 cells play an important role *via* producing interleukin (IL)-17A, which enhances bone resorption by bone degrading osteoclasts cells as reviewed by Tang et al. and Wu et al. in the context of postmenopausal osteoporosis (PMO), psoriatic arthritis (PsA) rheumatoid arthritis (RA), and axial spondylarthritis (axSpA). From their discussion, it appears that targeting the immune-regulatory molecules involved in the modulation of osteoimmune response may be a feasible approach to several inflammatory bone diseases. The role of T-cell protein tyrosine phosphatase (TCPTP), a protein tyrosine phosphatase immune-modulator in osteoporosis has been exhaustively reviewed by Wang et al. Another T cell regulatory molecule butyrophilin (Btn2a2), a transmembrane protein that suppresses the differentiation and fusion of osteoclasts and reduces the bone resorption process, has been demonstrated by Frech et al. Altogether, these studies highlight the significance of T cells and their associated molecules in regulating osteoclast differentiation with potential applications in osteoimmunology.

The potential of the immune system to regulate bone cells appears to be much more complex as besides T cells, other immune cells are also known to influence the bone remodeling and fracture healing process. Using single cell RNA sequencing (scRNA-seq) Zhang et al. demonstrate that the frequency of B cells is significantly reduced in the early stage of the fracture healing process. This study indicates that B cell is a crucial regulator of the fracture healing process *via* inhibiting excessive regeneration of bone by producing various osteoblast inhibitors. On the other hand, a pioneering study by Sapra et al. is the first to show that IL-10 producing regulatory B cells (Bregs) possess anti-osteoclastogenic properties. Importantly, this study also revealed that reduction in the frequency of Bregs and their reduced tendency to produce IL-10 enhances bone loss in a mouse model of osteoporosis. This study opens novel avenues in the field of cellular immunotherapy for the treatment and management of osteoporosis. Additionally, Breedveld et al. revealed that IgA autoantibodies, by promoting the release of IL-6 and IL-8 by immune cells and osteoclasts, enhance bone resorption in RA patients. Thus, targeting the interactions between IgA and its FcαRI receptor could be a promising therapeutic target in the treatment of RA. Altogether these studies highlight the growing importance of adaptive immunity in modulating bone health.

## Role of Innate Immune Cells in Skeletal Homeostasis

Accumulating evidence suggests that both the innate and adaptive immune system contributes to inflammatory bone diseases including osteoporosis. Innate immune cells by producing various inflammatory modulators contribute to the pathogenesis of osteoporosis. This group of immune cells, along with modulating osteoclastogenesis, also exhibits the potential of transdifferentiating into multinucleated osteoclasts. Thus, highlighting the importance of innate immune cells in the context of osteoporosis, Saxena et al. review the role of different innate immune cells in osteoporosis in a detailed systematic manner, thereby adding strength to the emerging field of Immunoporosis. Furthermore, to emphasize the role of macrophages in inflammatory bone loss conditions such as osteoarthritis (OA), Thomson and Hilkens comprehensively review the involvement of macrophages in disease progression. Activation of macrophages and osteoclastogenesis is a hallmark of osteolysis and thus can be targeted by the application of local platelet rich fibrin (PRF). Kargarpour et al. demonstrated that PRF has anti-inflammatory potential by mitigating macrophage function and thus exhibits the ability to reduce osteoclastogenesis.

## Implications of Chronic Inflammation on Bone Health

Emerging clinical and molecular evidence suggests that local and systemic inflammation can trigger osteoporosis. Among several conditions, psoriasis (Pso) showed an increased risk of osteoporosis mainly due to the deficiency of vitamin D and chronic inflammatory conditions. Of note, few studies have revealed the close functional association between vitamin D and IL-33/ST2 signaling pathway in the bone remodeling process (5, 6). Based on this close functional link, De Martinis et al. reviewed their crosstalk in the pathogenesis of bone and skin pathologies (7). Chronic inflammation that increases osteoclastogenesis also adversely impacts adipogenesis by increasing adipocyte population in lieu of osteoblasts from the mesenchymal stem cells (MSC). To highlight the significance of chronic inflammation in regulating the adipogenesis process, Furesi et al. demonstrate that higher levels of pro-inflammatory cytokines viz. IL-17 suppresses the adipogenesis process. By employing a bioinformatics tool, Wang et al. report that upregulation of chronic inflammation leads to intervertebral disc (IVD) degeneration and discogenic pain. Further analysis shows that infiltration of CCR7^+^CD163^+^ macrophages and Tregs is significantly enhanced in the degenerative IVDs. Various anti-inflammatory agents are currently being employed in chronic inflammation of which glucocorticoid (GC) is the first-line anti-inflammatory agent that acts *via* glucocorticoid receptor (GR). Upon binding of GCs, GR dimerizes is essential for aiding the anti-inflammatory potential of GCs. In contrast to the beneficial effects of GR dimerization, Hachemi et al. report on the adverse effects of GR dimerization on the fracture healing process and thus establish the detrimental effect of GCs and GR on fracture healing. The life cycle of osteoblasts and osteoclasts is regulated by key modulators of apoptosis viz., Fas-ligand (FasL) which is a member of the TNF superfamily and plays a fundamental role in the healing of extraction sockets. Alccayhuaman et al. further support the notion that FasL is necessary for bone regeneration and the healing process and showed a significant reduction in bone volume over tissue volume (BV/TV) in a study performed using FasL knock out mice. The involvement of the immune system is now also identified in very rare bone complications like aseptic implant failure (AIF) and periprosthetic joint infections (PJI). It was observed that the expression of immunoregulatory markers viz. sCD28, cCD80, sCTLA4, and sBTLA is significantly altered in the joints of AIF and PJI patients compared to controls and thus could be employed as a promising diagnostic marker for both AIF and PJI (Jubel et al.).

## Potential Therapies to Alleviate Bone Health

Tissue engineering methods utilize progenitor cells as a potential therapy for the treatment of several diseases. Studies demonstrated that chondrogenically primed human MSC (hMSCs) reiterate the methodology of endochondral ossification and in forming mature bone. To further translate this MSCs based approach in bone formation, Fahy et al. clearly show that implantation of MSCs in an immunocompetent xenogenic model promotes endochondral ossification and provides in-depth mechanistic insights into the osteoimmunological processes that regulate the regeneration of bone and homeostasis.

## Conclusion

The field of “Osteoimmunology” will provide novel etiologic insights into skeletal system-associated ailments in the near future. This might help in the design of therapeutic strategies for the management of various inflammatory bone pathologies with better translational potential. We (the editors) strongly believe that each article published under this Research Topic on “*Recent Advances in Basic and Translational Osteoimmunology*” will help in the discovery of new molecular candidates and pathways and unravel this complex yet important connection between the bone and immune system.

## Authors Contributions

All the authors have made extensive, direct, and intellectual contributions to the present work and approved it for publication.

## Conflict of Interest

The authors declare that the work has been conducted in the absence of any financial and commercial relationships that could lead to a potential conflict of interest.

## Publisher’s Note

All claims expressed in this article are solely those of the authors and do not necessarily represent those of their affiliated organizations, or those of the publisher, the editors and the reviewers. Any product that may be evaluated in this article, or claim that may be made by its manufacturer, is not guaranteed or endorsed by the publisher.
